# Nurses' Trials—No Drudgery

**Published:** 1887-02-26

**Authors:** 


### Words of Consolation.

[Communications for this column should be addressed, "Chaplain," care of the Editor of The Hospital, who will gratefully welcome any suggestions or inquiries from matrons, nurses, and the staff generally.]

XXII.—Nurses' Trials—No Drudgery.

If nurses will only aim at a high standard in devotion, however much at times they may fall short of it, there will be no fear of their service degenerating into drudgery. But if, on the other hand, their calling is regarded as purely secular, or only obeyed as a means of gaining a livelihood; then, almost more than any other, is it apt to become a kind of slavery. The amount of unpleasant, disagreeable, not to say disgusting, details with which nurses have to become familiar, and then to steel their hearts to endure, is terrible to contemplate from one point of view. These are enough at first to make many a woman's blood run cold who is naturally brave, and yet is, as she ought to be, femininely sensitive. She must, therefore, in her hospital life accept one of two alternatives. She must (1) either harden herself to sights and pains and disagreeable details which shock her feelings at the outset, and, becoming callous and indifferent to these experiences, become also the slave of the circumstances which surround her. Or (2) she may accept her high calling in Christ Jesus. She may look upon all that is given her to do as to be done for Him. She may regard the meanest, dirtiest, or most unwelcome work as never beneath being counted a sacrifice for One who condescended to be laid in the manger, and amongst unclean animals, for her sake. So, taking the highest view of the lowest work, she will never permit the least pleasant portions of her duty to degenerate into drudgery; for they will be continually elevated and sanctified by the thought, "Inasmuch as ye have done it unto one of the least of these my brethren, ye have done it unto me" (Matt. xxvi, 40). There can be no drudgery, no slavery, when such a motto as this hangs suspended over your daily exertions. The words should govern the whole life, of course. But they may be called to mind for your great consolation each time any feeling of disgust or of shrinking creeps over you; for what woman with a woman's heart can be a stranger to these recoils of the spirit from burdens so calculated to breed disgust? "Ye have done it unto me." You can carry the words far down into those very trying details of your work —let us say—the dressing of sores; the emptying and washing of vessels; the making of beds; the care and cleansing of linen and clothes; the ministration of food and medicine, when the very sight or smell of either may be repulsive; the listening to murmurs and complaints; the wearisome tension of watching symptoms. Oh! surely the aggregate of these must affect your heart and life for all eternity one way or the other, however slowly or imperceptibly the impression is being made. They must harden you into, perhaps, a very creditable slave of circumstances, or soften you into a tender servant of Jesus Christ.

Strive then to make your calling and election sure in the service of Christ and His members, and there will be no drudgery. Temptations to regard certain details as such will be frequent. But as often as these arise, and you feel a difficulty in working in a Christian spirit, say, "Lord, what I could not for others I can do for Thee; for Thou lovedst me and gavest Thyself for me, that I might give myself back to Thee in these humble yet heavenly offices for Thy suffering members."

(*To be continued.*)

